# Comprehensive Comparison of Front- and Back-Illuminated Single-Photon Avalanche Diodes in 110 nm Standard CMOS Image Sensor Technology

**DOI:** 10.3390/s26051664

**Published:** 2026-03-06

**Authors:** Doyoon Eom, Won-Yong Ha, Eunsung Park, Jung-Hoon Chun, Jaehyuk Choi, Woo-Young Choi, Myung-Jae Lee

**Affiliations:** 1 Department of Electrical and Electronic Engineering, Yonsei University, Seoul 03722, Republic of Korea; 2 Institute of Microengineering, École Polytechnique Fédérale de Lausanne (EPFL), 2002 Neuchâtel, Switzerland; 3 Department of Semiconductor Systems Engineering, Sungkyunkwan University, Suwon 16419, Republic of Korea; 4 TruPixel, Inc., Daejeon 34138, Republic of Korea

**Keywords:** back-illuminated (BI), dark count rate (DCR), CMOS image sensor (CIS), front-illuminated (FI), photon detection probability (PDP), single-photon avalanche diode (SPAD), standard CMOS technology, timing jitter

## Abstract

**Highlights:**

**What are the main findings?**

- Front-illuminated (FI) and back-illuminated (BI) SPADs fabricated using the same 110 nm CMOS image sensor (CIS) process and identical front-end-of-line (FEOL) structures exhibit distinctly different spectral responses, governed solely by the illumination direction and back-end-of-line (BEOL) design.
- The BI SPAD provides enhanced near-infrared photon detection probability (PDP), while maintaining comparable dark count rate (DCR) and timing jitter performance to the FI SPAD.

**What are the implications of the main findings?**

- We demonstrate that CMOS-based SPAD performance can be effectively optimized through illumination and BEOL engineering without modifying the junction structure or doping profiles.
- We provide practical design guidelines for selecting FI or BI SPAD architectures according to wavelength sensitivity and timing requirements in LiDAR and related applications.

**Abstract:**

This paper presents a process-controlled study of illumination engineering in single-photon avalanche diodes (SPADs) fabricated in a 110 nm standard CMOS image sensor (CIS) technology. Front-illuminated (FI) and back-illuminated (BI) SPADs were implemented with identical front-end-of-line (FEOL) structures, including the junction and guard-ring configurations, enabling the isolation of the effects of illumination direction and back-end-of-line (BEOL) configuration without modifying the junction structure. Through TCAD simulations and comprehensive experimental characterizations, including current–voltage, light-emission, dark count rate (DCR), photon detection probability (PDP), and timing-jitter measurements, we systematically analyze the performance trade-offs introduced by the BI configuration. The BI SPAD exhibits enhanced near-infrared PDP and a broader spectral response due to its deeper absorption region and the incorporation of a metal reflector, while maintaining identical avalanche characteristics, as evidenced by an unchanged 72 ps full-width-at-half-maximum (FWHM) timing jitter. However, the backside illumination increases the diffusion tail, indicating a trade-off between near-infrared sensitivity and diffusion-related timing performance. These results provide design guidelines for optimizing SPAD performance through illumination-direction and BEOL engineering while preserving the FEOL design and demonstrate a useful approach for SPAD integration in standard CMOS technology.

## 1. Introduction

Single-photon detectors are highly required in several applications such as imaging and ranging, quantum communications and networks, biology, chemistry, astronomy, etc. [1,2,3,4,5,6,7,8,9,10,11,12]. In particular, for light detection and ranging (LiDAR) and time-of-flight (ToF) applications like autonomous vehicles and augmented reality/virtual reality (AR/VR) systems [13,14,15,16,17,18], the key requirement is a single-photon avalanche diode (SPAD) with high detection efficiency in near-infrared (NIR) wavelengths implemented in standard CMOS technology, which enables monolithic integration with circuitry and consequently cost-effective, compact, and high-resolution LiDAR sensors.

In CMOS technology, front-illuminated (FI) SPADs have been widely investigated and manufactured for various applications [19,20,21,22]. However, conventional CMOS-based FI SPADs suffer from inherent limitations due to the metals and/or dielectric stacks over the device, which reduce the photon transmission and thus the effective photon absorption in the active region. In addition, above all, FI SPADs exhibit intrinsically low detection efficiency at the NIR region because the PN junctions formed in CMOS technology are quite shallow, about 200 nm ~ 2 µm in general, while the NIR light has a much longer penetration depth, over 10 µm, in silicon. To overcome these limitations, back-illuminated (BI) SPADs have emerged as promising candidates for NIR applications. By allowing incident light to enter from the backside of the substrate, BI SPADs can exploit deeper absorption regions in silicon and achieve higher detection efficiency at longer wavelengths. In addition, BI configurations offer an increased fill factor by avoiding optical obstruction from back-end-of-line (BEOL) layers. Thanks to these advantages, CMOS-based BI SPADs have been reported for NIR-sensitive applications [23,24,25,26]. Nevertheless, the use of BI architectures may cause a decline in timing performance due to deeper photon absorption and carrier diffusion. In addition, despite the growing interest in BI SPADs, to the best of our knowledge, no prior work has reported a direct comparison between FI and BI SPADs fabricated using the same foundry CMOS process while maintaining the identical front-end-of-line (FEOL) structure. Such a comparison is crucial to confirm the influence of the illumination direction and BEOL design on device performance. In previous studies, differences between FI and BI SPADs were affected by variations in fabrication technology, device structure, or doping profiles, making it difficult to reveal the intrinsic impact of the illumination direction and BEOL design.

In this work, we present FI and BI SPADs fabricated using a 110 nm standard CMOS image sensor (CIS) technology, i.e., via a foundry service. Both devices share identical junction structure and geometry, doping profiles, and guard-ring (GR) configuration, allowing a fair and process-independent comparison. The SPADs are investigated through technology computer-aided design (TCAD) simulations and experimentally evaluated in terms of current–voltage characteristics, light emission test (LET), dark count rate (DCR), photon detection probability (PDP), and timing jitter. Through this comprehensive comparison, we provide practical and straightforward insights into the performance trade-offs between FI and BI SPADs and assess their suitability for several applications operating at different wavelengths.

## 2. Materials and Methods

**Figure 1:**
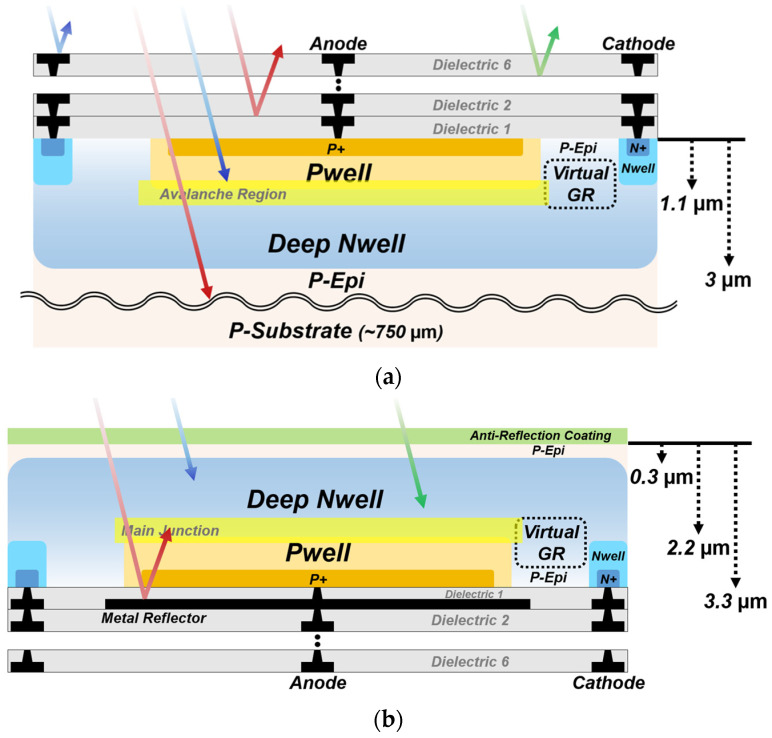
Cross-sections of the SPADs with different illumination configurations: (**a**) FI SPAD; (**b**) BI SPAD.

Figure 1 shows the cross-sections of the FI and BI SPADs with different illumination configurations. The FI SPAD features a circular avalanche region formed at the junction between the Pwell (PW) and the deep Nwell (DNW). The circular geometry is adopted to minimize local electric field crowding at the device periphery and to ensure a uniform electric-field distribution at the active region. A virtual GR is employed around the avalanche region to prevent premature edge breakdown (PEB). This structure utilizes retrograde doping of the DNW, which reduces the doping concentration at the periphery of the planar junction, thereby relaxing the electric field at the junction edge. The diameter of PW is 10 µm, while the widths of virtual GR and the surrounding Nwell (NW) are 2 µm and 1 µm, respectively. The depth of the avalanche region is approximately 1.1 µm, and the DNW extends to a depth of about 3 µm. Above the active region, multiple dielectric and metal stacks partially reflect incident photons in the FI configuration, thereby limiting photon absorption within the active region. The BI SPAD shares the identical FEOL structure with the FI SPAD, but it is vertically inverted as shown in Figure 1b. After backside thinning, the total wafer thickness is reduced to about 3.3 µm, corresponding to a remaining P-epi layer thickness of about 300 nm, while the P-substrate thickness in the FI SPAD is about 750 µm without backside thinning. An anti-reflection coating composed of a multi-layer dielectric stack is deposited on the backside to reduce optical reflection and enhance optical coupling efficiency. In contrast to the FI structure, where photons must pass through the dielectric stack before reaching the avalanche region, the BI SPAD receives incident photons directly from the backside, avoiding the BEOL layers in the optical path. Furthermore, the deeper avalanche region and the metal reflector, consisting of metal 1 under the device in the BI SPAD, can contribute to improved detection efficiency in the NIR region [27].

**Figure 2:**
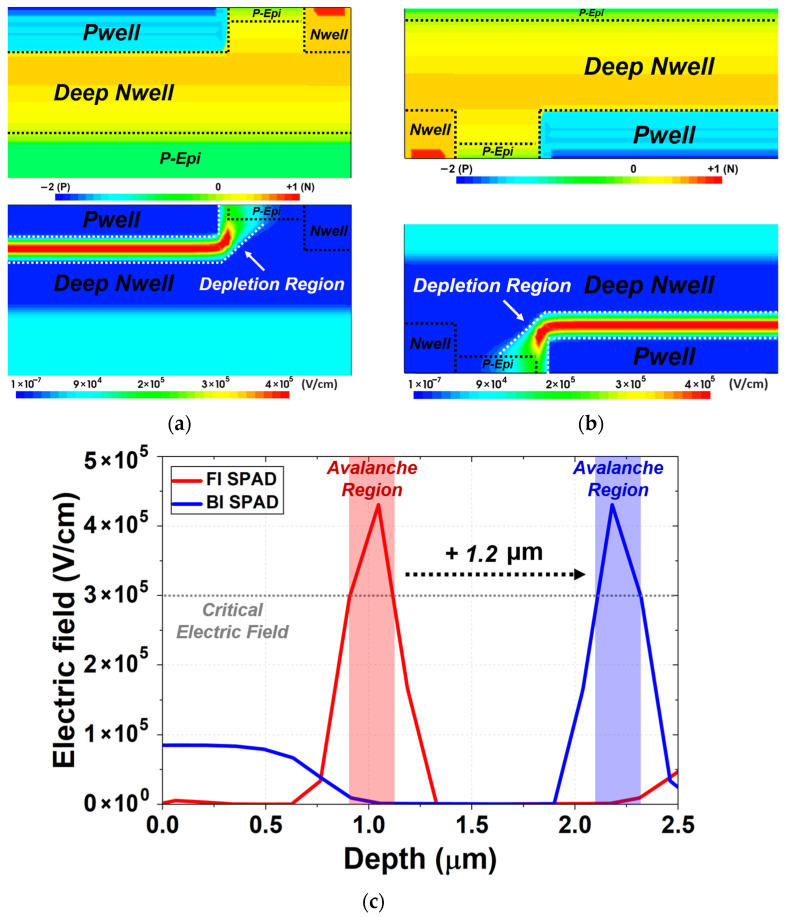
TCAD simulation results of the doping-concentration distributions presented in normalized scale and the corresponding electric-field distributions of the (**a**) FI SPAD and (**b**) BI SPAD. (**c**) Electric-field profiles of the FI and BI SPADs.

Figure 2 presents the TCAD simulation results of the FI and BI SPADs, including the doping concentration and electric-field distributions. In both devices, the PN junction is formed between the PW and the DNW, while the DNW exhibits a gradual vertical doping gradient toward the substrate as shown in Figure 2a,b. The P-epi layer is relatively lightly doped and extends beneath the DNW region, serving as the background region in both structures. The avalanche region follows the contour of the PW/DNW junction and extends laterally toward the PW edge, reflecting the planar geometry of the junction. In both SPADs, the electric field is uniformly formed across the planar junction, and it rapidly decreases within the deeper DNW and P-epi regions, indicating stable avalanche operation without edge breakdown. Although the overall electric-field distributions are identical in both SPADs, the BI SPAD exhibits a deeper avalanche region from the surface due to its back-illumination configuration. This deeper avalanche region enhances the absorption of long-wavelength photons and improves carrier-collection efficiency. Figure 2c compares the electric-field profiles of the FI and BI SPADs. The avalanche region forms near 1 µm in the FI SPAD, whereas in the BI SPAD it appears beyond 2 µm, corresponding to a shift of 1.2 µm relative to the FI structure. In both devices, the peak electric field exceeds the critical electric field required for avalanche multiplication, indicating successful formation of the multiplication region.

## 3. Results and Discussion

### 3.1. Current–Voltage Characteristics

**Figure 3:**
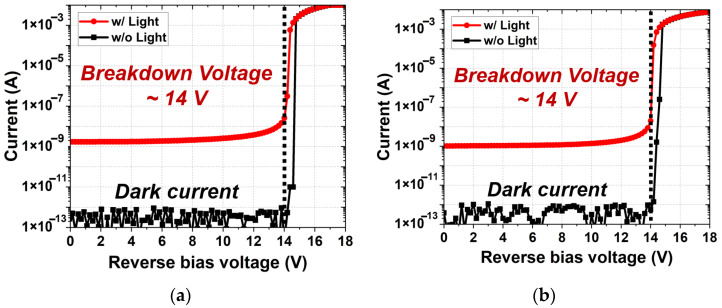
Current–voltage characteristics of the SPADs under the dark and illuminated conditions, showing avalanche breakdown behavior: (**a**) FI SPAD; (**b**) BI SPAD.

The current–voltage characteristics of the FI and BI SPADs were measured under dark and illuminated conditions, as shown in Figure 3. During the measurements, a reverse bias voltage was applied to the cathode while the anode was grounded. The substrate was grounded in accordance with the standard CMOS process environment, ensuring stable operation of the underlying CMOS devices. Both devices exhibit an extremely low dark current, below the picoampere level, prior to the avalanche breakdown. This indicates effective suppression of a leakage path in the depletion region. As the reverse bias approaches the breakdown voltage (*V_br_*), the current begins to rise gradually and then increases sharply once the avalanche-multiplication process is triggered. This abrupt transition reflects the extremely high multiplication gain of the SPADs. The FI and BI SPADs demonstrate nearly identical *V_br_* of approximately 14 V, indicating that the electric-field profiles and junction curvature in the multiplication region are well matched between the two structures.

**Figure 4:**
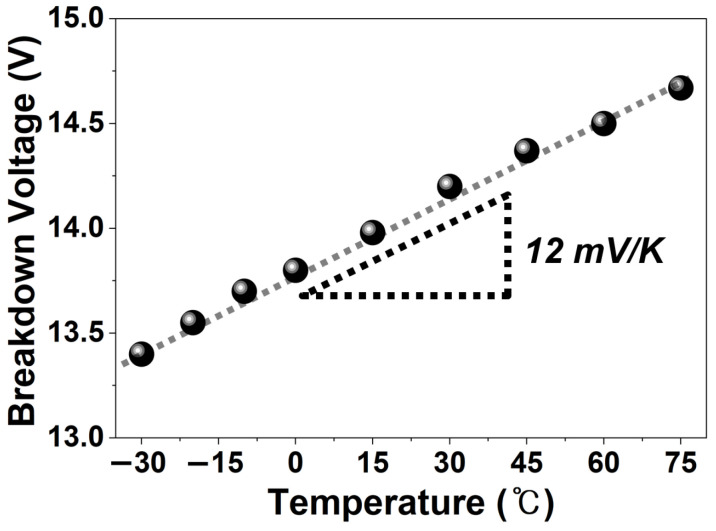
Temperature dependence of the breakdown voltage of the BI SPAD.

Figure 4 illustrates the temperature dependence of the *V_br_* of the BI SPAD over a temperature range from −30 to 75 °C. The result shows that the *V_br_* increases linearly with temperature, and this behavior is attributed to enhanced lattice vibrations at elevated temperatures. As the temperature rises, carriers in the depletion region experience more frequent scattering, which reduces their ability to gain sufficient energy for impact ionization, thereby lowering the avalanche probability [28]. Consequently, a higher electric field is required to initiate avalanche breakdown, leading to an increase in the *V_br_*. The measured temperature coefficient of *V_br_* is about 12 mV/K.

### 3.2. Light Emission Test (LET)

**Figure 5:**
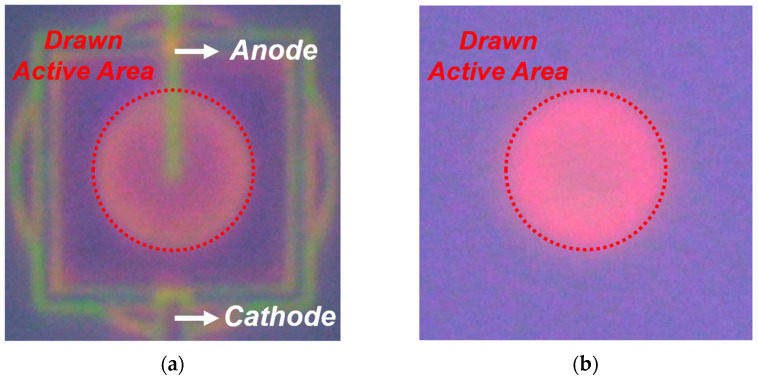
LET results of the SPADs at *V_ex_* = 3 V, visualizing avalanche emission across the active area: (**a**) FI SPAD; (**b**) BI SPAD.

To visually assess the effective active area and avalanche intensity, LET was performed at an excess bias voltage (*V_ex_*) of 3 V. LET exploits the phenomenon in which some hot carriers generated during avalanche breakdown recombine and emit photons. During this process, visible light is emitted in silicon, enabling the identification of regions contributing to avalanche multiplication using a visible camera, as shown in Figure 5. A low-intensity white LED integrated into the probe station was used solely for device probing and die-level alignment and did not contribute to avalanche generation. The emission is continuously generated under a steady bias. The emitted light was recorded using a conventional visible camera in standard video mode with the exposure time fixed at 1/30 s.

In the case of the BI SPAD shown in Figure 5b, the BEOL is located beneath the device, making it difficult to directly observe the BEOL configuration. Nevertheless, both SPADs exhibit uniform light emission across the entire drawn active area, indicating the absence of PEB [29]. The difference in the emitted light color is attributed to the junction depth. In the BI SPAD, photons generated at the junction propagate through a thicker silicon layer, which absorbs shorter (blue) wavelengths, resulting in a red-shifted emission as observed in Figure 5b.

### 3.3. Dark Count Rate (DCR)

**Figure 6:**
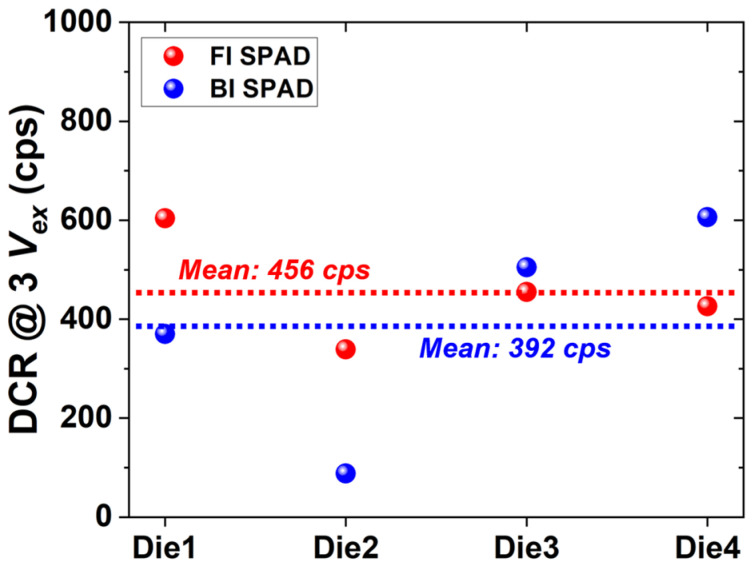
DCR of the FI and BI SPADs measured at *V_ex_* = 3 V, showing die-to-die variation and mean values.

Thermally or field-generated carriers can trigger an avalanche process, resulting in false detection pulses. DCR is the average number of output pulses from a SPAD per second when no incident photon is present on the SPAD. The DCR characteristics of the FI and BI SPADs were measured with quenching resistors connected in series to each SPAD anode, and the mean values obtained from four dies were used to account for die-to-die variation. All measurements were performed under the identical biasing condition, the excess bias voltage of 3 V, to ensure a consistent comparison between the FI and BI SPADs. As shown in Figure 6, the BI SPAD exhibits a slightly lower mean DCR compared to the FI SPAD. The mean DCR values of the FI and BI SPADs are 456 cps and 392 cps, respectively, at 3 *V_ex_*. This result indicates that the additional post-processing steps for the back-illumination, such as backside thinning, do not introduce a noticeable degradation in noise performance. The comparable DCR levels between the two structures can be attributed to the isolated SPAD design, where the avalanche region is electrically isolated from the substrate, thereby minimizing substrate-induced noise contributions.

**Figure 7:**
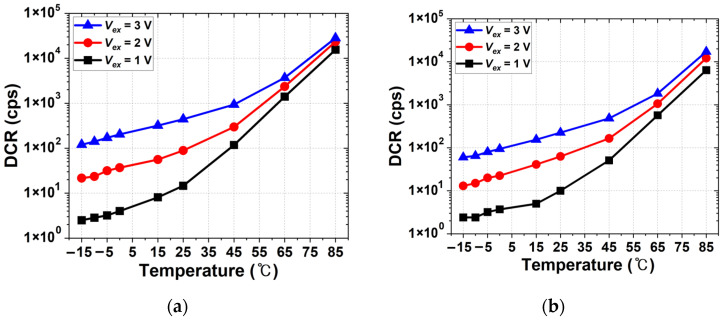
Temperature dependence of the DCR of the SPADs measured at different excess bias voltages: (**a**) FI SPAD; (**b**) BI SPAD.

Figure 7 shows the temperature dependence of the DCR of both devices measured at different excess bias voltages. Both SPADs exhibit similar trends as the temperature increases. The DCR increases approximately exponentially with temperature, which is characteristic of thermally activated carrier generation. At lower temperatures, the DCR increases gradually, whereas a much steeper increase is observed at higher temperatures. In addition, higher excess bias voltages result in increased DCR over the entire temperature range, which is attributed to the enhanced electric-field strength in the avalanche multiplication region. The FI and BI SPADs exhibit nearly identical temperature slopes under each bias condition, suggesting that their temperature-dependent noise behaviors are fundamentally similar.

**Figure 8:**
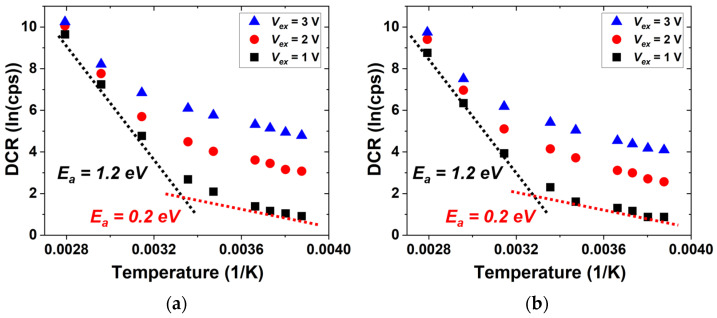
Arrhenius plots of the DCR at different excess bias voltages and extracted activation-energy values: (**a**) FI SPAD; (**b**) BI SPAD.

Figure 8 presents the Arrhenius plots derived from the data in Figure 7, along with the extracted activation-energy (*E_a_*) values. The extracted *E_a_* values are nearly identical for the FI and BI SPADs. Two distinct linear regions can be observed in the Arrhenius plots, indicating different dominant noise mechanisms in the low and high temperatures. The lower activation energy of 0.2 eV observed at low temperatures is consistent with trap-assisted tunneling processes associated with defect levels close to the band edges. In contrast, the higher activation energy of 1.2 eV at high temperatures, which is higher than the bandgap energy of silicon, 1.12 eV, implies that the thermal-generation process becomes dominant in this range. This result indicates that the defect density is low so that both devices achieve good DCR performance.

### 3.4. Photon Detection Probability (PDP)

**Figure 9:**
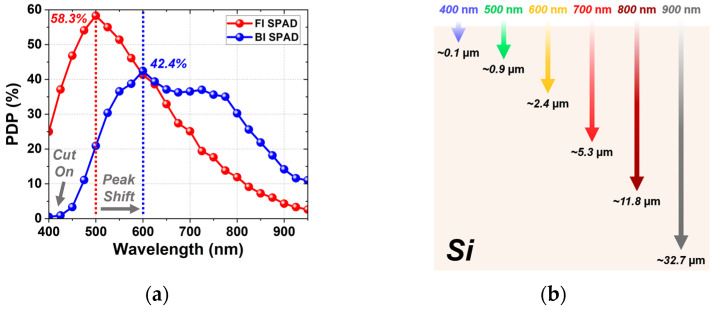
PDP spectra of the FI and BI SPADs at *V_ex_* = 3 V, together with the optical penetration depths of different wavelengths in silicon: (**a**) PDP spectra from 400 to 950 nm. (**b**) Optical penetration depths in silicon.

PDP is the probability that a single incident photon arriving at the active area of the SPAD triggers a pulse. Figure 9 shows PDP characteristics of the FI and BI SPADs, together with the optical penetration depths of different wavelengths in silicon [30]. The PDP was measured over the wavelength range from 400 to 950 nm at 3 *V_ex_*. As shown in Figure 9a, the FI SPAD exhibits a peak PDP of 58.3% at 500 nm. This result corresponds well to the optical penetration depth of silicon in Figure 9b, as light at 500 nm penetrates about 0.9 µm into silicon, which closely matches the junction depth of the FI SPAD. In the NIR region, however, the FI SPAD shows a reduced PDP because its shallow junction depth limits the absorption of long-wavelength photons, and the absence of a metal reflector prevents efficient photon recycling within the device. In contrast, the BI SPAD exhibits a broader spectral response and achieves a peak PDP of 42.4% at 600 nm. This wavelength corresponds to an optical penetration depth of about 2.4 µm, which is consistent with the avalanche-region depth of the BI SPAD, approximately 2.2 µm, as can be seen in Figure 1b and Figure 2c. In addition, the PDP of the BI SPAD slightly increases again near 700 nm owing to the metal reflector beneath the active region, which enhances photon absorption through internal reflection. These trends indicate that the BI-induced PDP enhancement is not limited to the NIR region but extends into the red-visible range, with the BI SPAD exhibiting higher sensitivity than the FI SPAD above approximately 600 nm.

At shorter wavelengths near 400 nm, however, the BI SPAD shows a pronounced cut-on characteristic in PDP. This behavior originates from the remaining P-epi layer with a thickness of about 0.3 µm. Because light at 400 nm penetrates only about 0.1 µm into silicon, most photons are absorbed within the P-epi layer. The photon-generated carriers in the region cannot reach the avalanche region due to the potential barrier between the P-epi and the DNW, and therefore do not contribute to any avalanche triggering. Consequently, the PDP decreases sharply in the short-wavelength range. This also indicates that extending the BI configuration toward shorter wavelengths requires careful management of surface carrier losses.

**Figure 10:**
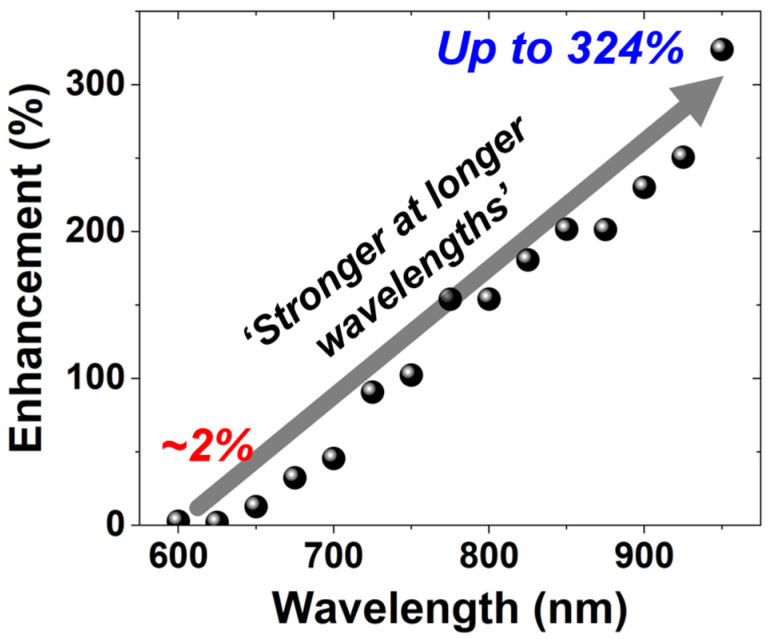
Wavelength-dependent PDP enhancement of the BI SPAD over the FI SPAD.

Figure 10 shows the wavelength-dependent PDP enhancement of the BI SPAD compared to the FI SPAD. The enhancement is only about 2% near 600 nm but increases significantly with wavelength, reaching up to 324% at 950 nm. These results clearly demonstrate that the BI structure is particularly effective at improving detection efficiency in the longer-wavelength region. Overall, these results indicate that the PDP characteristics of the FI and BI SPADs are primarily governed by the avalanche-region depth and illumination configuration. As a result, the two devices exhibit complementary spectral responses, with the FI SPAD optimized for visible-wavelength detection and the BI SPAD providing enhanced sensitivity in the red-to-NIR region. Such wavelength-dependent behavior highlights the suitability of each structure for different applications depending on the target spectral range.

### 3.5. Timing Jitter

**Figure 11:**
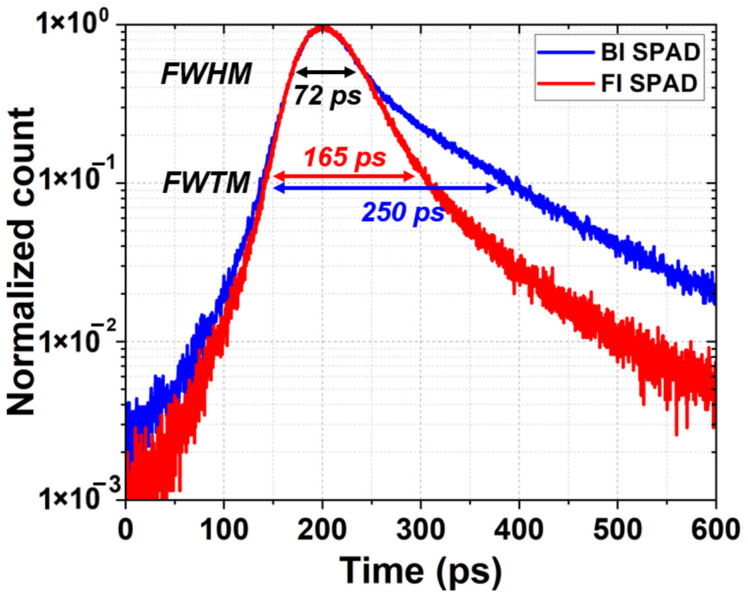
Timing jitter characteristics of the FI and BI SPADs at 940 nm and *V_ex_* = 3 V, showing the FWHM and FWTM of the temporal response.

Timing jitter is the uncertainty or variation in the time difference between the actual arrival of a photon at the SPAD’s active area and the generation of the corresponding output pulse. Timing jitter was characterized using the time-correlated single-photon counting (TCSPC) method with a 940 nm picosecond pulsed laser, a wavelength commonly employed in LiDAR applications. Timing jitter is typically quantified by the full width at half maximum (FWHM) of the temporal response histogram. As shown in Figure 11, both the FI and BI SPADs exhibit excellent timing performance, achieving a FWHM of about 72 ps at the excess bias voltage of 3 V. This result indicates that the primary timing resolution of the devices, which is mainly governed by the drift time in the depletion region and avalanche build-up time in the avalanche region, is not significantly affected by the illumination configuration.

In addition to the FWHM, the full width at tenth maximum (FWTM) was also evaluated to assess the diffusion tail of the temporal response. The BI SPAD exhibits a larger FWTM of 250 ps compared to 165 ps for the FI SPAD. This increased FWTM value indicates a longer diffusion tail in the BI SPAD, which can be attributed to the increased carrier diffusion length resulting from the back-illumination with the presence of a metal reflector. In the present device structure, carrier transport contributing to avalanche triggering is primarily confined to the DNW region, while contributions from carriers generated in the substrate are strongly suppressed by the DNW/P-substrate junction. Therefore, the diffusion tail is governed mainly by optical propagation and absorption within the active transport region rather than by the substrate thickness itself. In the BI SPAD, the metal reflector increases the effective optical path length within this region, which enhances the diffusion transport and results in a larger FWTM. Although timing jitter was measured at 940 nm in this work, the same framework can be extended to interpret wavelength-dependent tail behavior. More specifically, the wavelength dependence of the diffusion tail can be understood by considering the relative relationship between the optical penetration depth and the avalanche-region depth. At shorter wavelengths, the optical penetration depth decreases, which may change the relative contributions of drift and diffusion transport and, in turn, affect the timing distribution [31].

From the application perspective, the identical FWHM values ensure that the BI SPAD maintains high timing resolution suitable for ToF and LiDAR systems, while the extended FWTM represents a trade-off associated with the PDP in the NIR region. Consequently, the timing jitter characterization results highlight a fundamental trade-off between NIR sensitivity and diffusion-related timing jitter.

Table 1 compares this work with previously reported SPADs fabricated in 110 nm CIS technology, while excluding additional process techniques so that the comparison reflects intrinsic device characteristics [19,32,33,34]. Relative to prior reports, this work shows competitive overall performance in terms of DCR, peak PDP, NIR PDP, and timing jitter while preserving a CMOS-compatible structure. In particular, the results confirm that illumination and BEOL engineering can effectively extend sensitivity toward longer wavelengths without a substantial penalty in timing performance. Although some previous studies report higher peak PDP under different operating conditions or wavelength targets, the present results demonstrate a well-balanced trade-off in overall performance.

## 4. Conclusions

In this work, FI and BI SPADs were fabricated and directly compared using the same 110 nm standard CIS technology. By maintaining identical FEOL structures, the intrinsic effects of illumination direction and BEOL configuration were clearly identified through both TCAD simulation and experimental measurements. The BI SPAD demonstrates comparable DCR and primary timing jitter performance to those of the FI SPAD, while achieving a broader spectral PDP response with enhanced sensitivity in the NIR region due to its deep avalanche region and the use of a metal reflector. Although the BI SPAD showed an increased diffusion-related timing tail, the FWHM remained unchanged, indicating that the fundamental timing resolution is preserved. In contrast, the FI SPAD showed higher PDP in the short-wavelength region, making it more suitable for short-wavelength photon detection. These results clearly verify a design trade-off between enhanced NIR sensitivity and timing tail behavior. Overall, this comparative study confirms the feasibility and practical advantages of implementing both FI and BI SPADs within a standard CMOS process and provides useful design guidelines for optimizing SPAD architectures toward specific applications.

## Figures and Tables

**Table 1:** Comparison of this work with prior 110 nm CIS SPADs without additional process techniques.

	This Work		[19]	[32]	[33]	[34]
Technology	110 CIS		110 CIS	110 CIS	110 CIS	110 CIS
Illumination	FI	BI	FI	FI	BI	FI
Junction	PW/DNW		PW/DNW	PW/DNW	PW/DNW	N+/HVPW
GR	Virtual		Virtual	Virtual	Virtual	Virtual
Active Area (μm^2^)	78.5		78.5	78.5	277	78.5
*V_br_* (V)	14		14	18	15.4	17.2
*V_ex_* (V)	3		3	3	2.6	5
DCR (cps/μm^2^)	5.8	5.0	4.59	0.4	45.1	12.6
Peak PDP @ *λ*	58.3% @ 500	42.4% @ 600	58% @ 500	64% @ 500	33% @ 600	73% @ 440
PDP at NIR	2.6% @ 950	11.02% @ 950	3.02% @ 940	5.5% @ 905	11.1% @ 905	2.57% @ 940
Jitter (ps) @ *λ*	72 @ 940	72 @ 940	71 @ 670	92 @ 850	-	79 @ 850

## Data Availability

The original contributions presented in this study are included in the article. Further inquiries can be directed to the corresponding author.

## Abbreviations

AR/VR	Augmented Reality/Virtual Reality
BEOL	Back-End-of-Line
BI	Back-Illuminated
CIS	CMOS Image Sensor
CMOS	Complementary Metal-Oxide-Semiconductor
DCR	Dark Count Rate
DNW	Deep Nwell
FEOL	Front-End-of-Line
FI	Front-Illuminated
FWHM	Full Width at Half Maximum
FWTM	Full Width at Tenth Maximum
GR	Guard Ring
LET	Light Emission Test
LiDAR	Light Detection and Ranging
NIR	Near-Infrared
NW	Nwell
PDP	Photon Detection Probability
PEB	Premature Edge Breakdown
PW	Pwell
SPAD	Single-Photon Avalanche Diode
TCAD	Technology Computer-Aided Design
TCSPC	Time-Correlated Single-Photon Counting
ToF	Time of Flight
